# Cross-cultural adaptation and content and semantic validation of the Difficult Intravenous Access Score for pediatric use in Brazil ^1^

**DOI:** 10.1590/1518-8345.1785.2920

**Published:** 2017-09-18

**Authors:** Márcia Helena de Souza Freire, Cristina Arreguy-Sena, Paula Christina de Souza Müller

**Affiliations:** 2 PhD, Adjunct Professor, Universidade Federal do Paraná, Curitiba, PR, Brazil.; 3 PhD, Adjunct Professor, Universidade Federal de Juiz de Fora, Juiz de Fora, MG, Brazil.; 4 MSc, RN, Universidade Federal do Paraná, Curitiba, PR, Brazil.

**Keywords:** Nursing Methodology Research, Validation Studies, Pediatric Nursing, Hospitalization, Catheterization Peripheral

## Abstract

**Objective:**

present the cross-cultural adaptation and content and semantic validation of the Difficult Intravenous Access Score for current use in Brazil.

**Method:**

cross-cultural adaptation and validation study, structured in six phases: initial translation, synthesis of translations, back-translation, assessment of documents by expert committee of specialized judges, pretest and presentation of the documents to the expert judges and to the author of the original instrument. Twenty health professionals were randomly recruited from a public hospital in the South of Brazil, working in pediatrics, in order to assess the agreement level with the variables in the instrument. In addition, a convenience sample of 30 pediatric patients was selected for the face validation of the same instrument. Cronbach’s alpha coefficient, simple and percentage frequencies, the Shapiro-Wilk and Fisher’s exact tests were used for the data analysis and reliability measures.

**Results:**

the cross-cultural adaptation phases were executed with totally clear translated variables, demonstrating satisfactory results in the content and semantic validation process.

**Conclusions:**

the Difficult Intravenous Access Score was adapted and its content and semantics were validated. External clinical validity, measuring equivalence and reproducibility analyses are needed.

## Introduction

The environment and workflow in child hospitalization, like in other hospital contexts, are permeated by hard technologies, excessive manipulations and invasive and painful procedures. The execution of the procures conspires towards the origin of fear, insecurity and anxiety, in the children as well as their relatives/companions, perpetuating the negative image children and young people hold of the hospital and hospitalization^1^ ^-^ ^2^. In that perspective, it is emphasized that hospitalized children and their relatives face difficulties to understand and assimilate the new scenario, reflecting in highly intense emotional responses to the care provided. This spectrum is further aggravated when the child is submitted to invasive and painful procedures, mainly in case of distancing among the child/relative/companion, and to a blunt invasion of the limits of their privacy and physique^3^ ^-^ ^4^. 

In that sense, Peripheral Intravenous Puncture (PIP) is a common nursing procedure in hospitals and health services. It is estimated that more than 35% of the patients submitted to hospitalization need PIP for medication and solution administration, implementing the clinical therapeutics established^5^. Likewise, many factors can interfere in the success of PIP in the pediatric population, and the clinical history of puncturing difficulties, malnutrition, antecedents of vascular traumas are examples of complicating factors for the establishment of PIP^6^. 

In view of the problem to predict the pediatric patients’ difficulty, considering the failure to establish PIP upon the first attempt, in 2008, a North-American medical team, in partnership with nurses from a pediatric emergency center, developed a score to quantify this difficulty, called the Difficult Intravenous Access Score (DIVA score)^7^.

The DIVA score consists of predictive variables, which are: visibility, palpability, age, prematurity and skin shade. Each variable is scored, and all scores are added up, indicating that children scoring 4 or higher will have a 50% higher probability of failure to establish PIP upon the first attempt^7^ ^-^ ^9^. The score was validated in the United States in 2011 and in Ireland in 2012, concluding that its use is fast and effective, producing information for the option to use adjuvant resources^8^ ^-^ ^9^. 

Nevertheless, to apply the DIVA score in the Brazilian reality, in coherence with the original tool, we aimed to propose a Portuguese version for current use in Brazil. In this article, we aimed to describe the cross-cultural adaptation and content and semantic validation of the Difficult Intravenous Access Score (DIVA score) for pediatric use in Brazil. 

The impact of using adjuvant resources, in this case the DIVA score, direct and positively influences the quality of the pediatric PIP procedure, as it can minimize negative psychological effects, considering the high stress during this procedure, for patients and relatives as well as for the health team. In addition, the resources are expected to lower the costs of the PIP procedure, as high rates of PIP failure entail high financial costs, expressed as: excessive spending on material, longer nursing work time, effort of the health team to solve the health problems and extended hospitalization^10^. 

## Method

The cross-cultural adaptation of health tools for use in Brazil requires the use of a method to achieve the equivalences between the language of origin and the target language. The content validity needs to be preserved, permitting the understandable application of the instrument in the new language, with consistent internal adaptation of the linguistics and culture, guaranteeing that the assessed impact of a disease or treatment is being described similarly in multinational trials^11^ ^-^ ^12^.

Hence, this study was designed based on the Cross-Cultural Adaptation and Validation Method, in line with the recommendations of the Guidelines for the Process of Cross-Cultural Adaptation of Self-Report Measures, which are six interdependent phases: initial translation, translation synthesis, back-translation, expert committee review, pretest and submission of documentation to the expert judges and author of the original tool^11^.

In the course of the process, which took place between February and October 2015 at a public hospital in the South of Brazil, the equivalences proposed by the Guideline were analyzed: semantic (equivalence in the meaning of the words), idiomatic (words peculiar to a language), experiential (situations represented in the original version that should be adapted to the cultural context of the target language) and conceptual (related to the concept validity) equivalences, which provide the validation of the instrument. It should be kept in mind that the variables, which are the instrument’s measures, need to be adapted to maintain the content validity in the different cultures. Hence, simply translating them is insufficient^11^ ^-^ ^12^. 

In the fifth phase, the pretest, two developments took place. First, the pretest of the cross-culturally adapted tool was applied to assess the clarity of its translated variables, involving 20 health professionals working in pediatrics (physicians, nurses, nursing technicians and auxiliaries). That distribution per professional category was unplanned but happened based on the availability of the professionals in the sector on the days the pretest was applied. Then, the pretest was applied to 30 children from the target group (n=30), using a convenience-based population sample, as the instrument was applied for validation instead of intervention purposes. Thus, no invasive intervention took place, but merely an ectoscopic evaluation of each children’s upper limbs, based on the criteria of the translated DIVA score. For the analysis of the data deriving from the application of the translated DIVA score to the 30 pediatric patients, simple description was used with absolute figures and percentages. In addition, the Shapiro-Wilk and Fisher’s exact statistical tests were applied, with the support of the Statistical Package for the Social Sciences (SPSS).

Specific tools were developed to register each phase of the process, allowing each segment involved to document its opinions in the methodological process, registering its observations. Approval for the project was obtained from the Research Ethics Committee of the Health Sciences Sector, Universidade Federal do Paraná, under Opinion No. 954.460. The premises of Resolution 466/12 were adopted, about the guidelines and regulatory standards for research involving human beings^13^. For the cross-cultural adaptation and validation of the original tool and further dissemination, formal authorizations were obtained from the original authors.

## Results

The methodological trajectory started with the initial translation by two bilingual translators with distinguished profiles, limited to the translation of the original tool from English to Brazilian Portuguese. In this phase, Translator 1, graduated in health, was familiar with the concepts analyzed, while Translator 2 had neither background in the area nor knowledge on the concepts analyzed. Each translator produced an independent version. This situation rests on the explanation that the absence of a background in health provides for a translation that can reflect the language the overall population uses, without the influence of the clinical perspective^11^ ^-^ ^12^. The instrument was named according to the translators’ different translators of the variable: Translator 1 described the title as *Escore de acesso intravenoso difícil -* DIVA score; and Translator 2 as *Difícil ponto de acesso intravenoso*.

In the second phase, after the assessment of the disagreements and equivalences between the translations resulting from the previous phase, both translators produced a consensus version, concluding the synthesis version of the translations^11^ ^-^ ^12^.

In the third phase, two translators without a background in health were chosen to elaborate the back-translation. They only had access to the consensus version synthesized in the previous versions of the process and each translator produced an independent back-translation. The native language of both translators was North-American English. Therefore, they back-translated the instrument from the target language (Brazilian Portuguese) to their mother tongue (North-American English). Like in the previous phase, the translators produced a consensus version. This back-translation phase corresponds to the verification process of the validity, and also guarantees that the translated version reflects the conceptual equivalence to the original version^11^ ^-^ ^12^.

In the fourth phase, the expert committee (n=10) consisted of two translators, two back-translators, four bilingual experts and two language professionals (one specialized in methodology and the other in linguistics).

Between translators T1 and T2, both of whom were proficient in the mother tongue of the original score, English, T1 was familiar with the concepts analyzed and had a background in health, with a view to promoting equivalence from the clinical perspective. T2, without a background in health, was not familiar with the concepts analyzed. The absence of a background in health provides for a translation that reflects the population’s current language instead of the clinical perspective. 

The back-translators chosen did not come from the health area, were not familiar with the concepts analyzed and did not have access to the original tool, but merely to the translated version synthesized in the translation phase of the process. Both were native speakers of North-American English and back-translated the tool from the target language (Brazilian Portuguese) to their mother tongue (North-American English).

**Figure 1:**
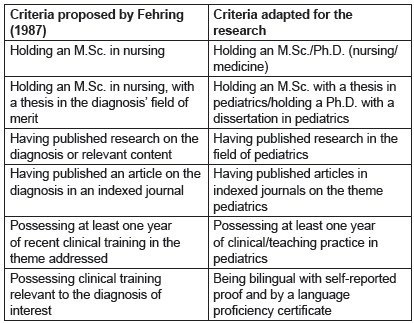
Classification of experts according to Fehring (1987) and adaptations by the authors. Curitiba, PR, Brazil, 2015

The methodology professional made the methodological decisions together with the linguist (responsible for analyzing the sentence structure and the meaning of the expressions), so as to obtain effective results and make decisions for the proposed equivalences. To select the bilingual experts, an instrument was used, adapted from the criteria to define an expert^14^, which is fundamental to choose experts, as described in Figure 1.

After the choice, all experts received the versions of the instrument to be adapted, as well as the instructions to apply the instrument and the possibilities to establish the score. Thus, they made decisions for the purpose of semantic equivalence between the original version and the target version, consolidating all versions of the instrument and supported the development of the pre-final version for the field tests.

In the fifth phase, the 20 professionals individually registered their agreement level with the clarity of the variables in the Portuguese version of the score on a Likert scale^15^. The answers on the Likert scale were numbered from 1 to 5, with 1 corresponding to “not clear at all” and 5 to “completely clear”. The participants could also register opinions concerning their answers in an exclusive area for comments. Among the participants, 7 (35%) were physicians, 2 (10%) nurses; 3 (15%) nursing technicians; and 8 (40%) nursing auxiliaries. One expected but noteworthy fact is that 65% (13) of the professionals who answered the pretest came from the nursing area, predominantly the secondary level, that is, nursing technicians and auxiliaries, adding up to 55% (11 professionals). And, as the prevalent sex of the participants, 17 were women (85%). It can be inferred that the participants presented significant professional baggage, as 55% (11 professionals) possessed more than 11 years of professional experience (Table 1). 

**Table 1:** Characteristics of health professionals who answered (n=20) the pretest questionnaire, Likert DIVA* score, according to sex, profession and length of experience. Curitiba, PR, Brazil, 2015

**Identification variables**		**Distribution**	
		**N**	**%**
**Sex**			
	**Female**	**17**	**85**
	**Male**	**3**	**15**
**Profession**			
	**Physician**	**7**	**35**
	**Nurse**	**2**	**10**
	**Nursing technician**	**3**	**15**
	**Auxiliary nurse**	**8**	**40**
**Length of experience**			
	**<12 months**	**5**	**25**
	**1 to 10 years**	**4**	**20**
	**11 to 20 years**	**6**	**30**
	**≥20 years**	**5**	**25**

*Difficult Intravenous Access

Next, following the recommendations in the Guideline, the pre-test was applied to 30 children from the target group (Table 2), a population-based convenience sample, as the score was applied for validation instead of intervention purposes. Hence, no invasive intervention was made, but mere ectoscopic assessment of each child’s upper limbs, based on the criteria of the translated DIVA score. Equal proportions of female and male children were assessed, and the majority (53%) was more than two years old (Table 2).

**Table 2:** Characteristics of the children (n=30) according to sex and age range, and of the criteria assessed with the support of the DIVA* *score*, according to visibility, palpability, age, prematurity and skin shade in the application of the pretest. Curitiba, PR, Brazil, 2015

Identification variables and DIVA* score **assessment criteria**		**Distribution**	
		**N**	**%**
**Sex**			
	**Male**	**15**	**50**
	**Female**	**15**	**50**
**Age range**			
	**≤12 months**	**13**	**44**
	**13-24 months**	**1**	**3**
	**≥24 months**	**16**	**53**
**Visibility**			
	**Visible**	**14**	**47**
	**Invisible**	**16**	**53**
**Palpability**			
	**Palpable**	**19**	**63**
	**Not palpable**	**11**	**37**
**Age**			
	**≥36 months**	**11**	**37**
	**12-35 months**	**6**	**20**
	**<12 months**	**13**	**43**
**Prematurity**			
	**Not premature**	**25**	**83**
	**Premature**	**5**	**17**
**Skin shade**			
	**Light**	**20**	**67**
	**Dark**	**10**	**33**

*Difficult Intravenous Access

As for the criteria assessed by means of the DIVA score, 16 (53%) children presented an invisible venous network, despite the application of a tourniquet to the inspected limb. What the palpability is concerned, 19 (63%) children had palpable veins, that is, detected through tactile inspection, using the digital pulp of the middle fingers and/or right and/or left indicators. 

With regard to the age in the convenience sample, according to the age groups in the DIVA score, there were children under 12 months of age (n=13; 43%), and this group obtained a higher DIVA score. The children in the age range between 12 and 35 months (n=6; 20%) received an intermediary DIVA score. The age group ≥36 months, on the other hand, did not score.

The criterion that scored highest on the DIVA score was prematurity. Being born prematurely, according to the original studies and validation of the DIVA score, corresponds to three points, to be added to the total score. Full-term children ≥3 years of age, then, did not score at all. In this pre-test, five (17%) premature children were assessed.

To measure the reliability among the health professionals’ opinions on the clarity of the translated score, the equivalence coefficient between the Brazilian Portuguese version and the North-American English version was assessed and Cronbach’s alpha coefficient was used^14^, with the support of the statistical software SPSS^®^.

Since the total score of the judges showed no normal distribution, based on the verification by means of the Shapiro-Wilk test, with a p-value < 0.0001 the dichotomization of the scores was considered with a view to using more appropriate statistical analysis for the sake of statistical reliability. In the dichotomized Likert scale, the alternative answers were treated, in view of their positive or negative tone, respectively, as: “unclear”, combining the levels “not clear at all” and “hardly clear”; and “clear”, combining the levels “clear”, “very clear” and “completely clear”. Hence, based on the new configuration of the clarity levels, we can assume that the score was completely reliable, as it was considered “clear” in 100% of the assessments. 

**Figure 2:**
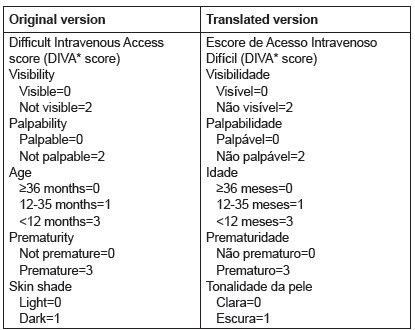
Original version of the DIVA score and version cross-culturally adapted to Brazilian Portuguese

The final phase of the cross-cultural adaptation and validation process of health instruments, the sixth phase, corresponded to the submission of all reports, forms and the final version of the instrument (Figure 2) to the expert committee and to the author of the original instrument.

*Difficult Intravenous Access

## Discussion

The cross-cultural adaptation of health instruments for application in a new country requires the use of a single method to achieve the equivalences between the language of origin and the target language. This refers to the need to maintain the content validity and, thus, permit the application of the instrument in the new language, with linguistic and cultural adaptation, guaranteeing that the assessment of the impact of a disease or treatment is described similarly in multinational trials^12^. 

Concerns with the use of instruments in health, translated exactly to other languages and cultural realities, emerged at the start of the 1990’s. This fact was precipitated by the use of these merely translated tools, or as a simple comparison with the back-translation, without equivalences to maintain the coherence with the original instrument, which culminated in improper uses and unsatisfactory results^16^.

In the analysis of the initial translation of the DIVA score, the experts agreed that Translator 1’s version, *Escore de acesso intravenoso difícil -* DIVA score*,* was closer to the professional practice. This version suggests the difficulty to establish intravenous access, differently from Translator 2, who suggested *Difícil ponto de acesso intravenoso*, marking or delimiting a specific place for the puncture, giving the impression of difficulties to access a specific location.

In case of difficult intravenous access, the professional, despite being qualified and experienced, is unable to establish the peripheral venous catheterization of pediatric patients after multiple attempts. This is not merely about manual skill, but also about physical aspects of the patient, psychological aspects (of professional and patient), use of material improper for the vein caliber, types of solutions to be infused - or established therapeutics -, among other factors^17^. Pediatric patients tend to be submitted to different attempts to establish the PIP by more than one professional. Thus, the difficulty to establish an administration route for solutions, mainly upon the first attempt, is a daily reality, especially at pediatric emergency services. Being a high-priority need for care delivery to critically ill and unstable children, it represents a cause of anguish and great stress in the team, the children and the relatives/companions when the PIP is not readily established^18^. 

After the translation, back-translation and assessment by the expert committee, the health professionals tested the clarity or reliability of the variables in pre-final version of the score in Brazilian Portuguese. Most participants were experienced in pediatric care, which can confirm the reliability of their assessment answers to the DIVA score, based on theoretical knowledge and accumulated practical skills. The score on the Likert questionnaire the health professionals completed went beyond an opinion of moderate reliability, as 100% of the participants confirmed the clarity of the instrument.

The assessed children equally represented the male and female sexes, and the majority was more than two years old. As regards the vulnerability to failure upon the first attempt, the children under 12 months of age showed to be less vulnerable, while the children between 12 and 35 months of age revealed intermediary failure according to the DIVA score. Nevertheless, the age group ≥36 months did not score, indicating that the age category for children over three years of age is of no weight in the total risk score of failure upon the first puncture. It can be inferred that, to predict failure upon the first PIP attempt, the age category is hardly significant as an isolated predictive variable. Nevertheless, when the other variables are calculated, a high risk can be found, even if the age-related score is nil. Similarly, it is observed that other clinical conditions influence the other variables, such as chronicity, the nutritional status and hydration. 

In the visibility criteria assessed by the DIVA score, it is highlighted that most children presented an invisible venous network, despite the application of a tourniquet to the visually inspected limb, a routine situation when a PIP is established and which causes stress in the puncture team. On the other hand, most children had palpable veins, that is, detected by means of tactile inspection. This scenario appoints the need for adjuvant resources though, with a view to the better location of the access, avoiding countless repeated punctures and, mainly, the children’s trauma.

Prematurity is the criterion that scored highest in the DIVA score. Premature birth, according to the original studies and validation of the DIVA, corresponds to 3 points, to be added to the total score. Thus, full-term birth can be considered a protection factor for the failure of puncture upon the first attempt. When analyzed alone, without considering the other items, prematurity alone results in a borderline score for first-attempt success. Only one additional point in the total score, related to any other variable, will put the child in an analysis context that does not dismiss the use of an adjuvant resource.

The clinical prediction score of the failure of first-attempt puncture can be used by phlebotomists, who work at laboratories were PIPs are executed in children, or even at outpatient clinic, where the blood collection is part of the daily work routine. The results evidenced that the DIVA score, translated to Brazilian Portuguese, is easy to apply in pediatric nursing care, and useful as a tool to qualify care in outpatient and hospital-based scenarios.

It is highlighted that the difficulties to develop this research were related to the choice and availability of the judges, experts and translators, and to the organization of the time for each of the six phases of the Cross-Cultural Adaptation and Validation Method.

## Conclusion

The concern with the PIP procedure and its difficulties derived from the daily practical experience at a pediatric emergency service, and determined the search for an alternative and/or predictive tool to support this painful and disturbing process, for the nursing team as well as the pediatric patients and their relatives/companions.

The possibility to adjust the DIVA score to the Brazilian reality, for use in pediatric care sectors, with a view to planning the care in an individualized, safe and responsible manner, centered on the wellbeing of the children and their relatives/companions, thus influencing the satisfaction and successful professional performance of the nursing team, were determinant factors towards their choice.

The Brazilian version of the DIVA score, called *Escore de Acesso Intravenoso Difícil*, was cross-culturally adapted with content and semantic validation or face validation, for current use in Brazil. It is an adjuvant instrument for the nursing diagnosis Risk for Vascular Trauma, as it defines pediatric patients who will have a 50% chance of failure upon the first attempt to establish the PIP. In addition, it favors a qualitative analysis of the care provided and permits concluding on the need to implement groups with expertise in pediatric vascular access, or on the use of technological devices that facilitate the visualization of the venous network. 

Consequently, it contributes to Brazilian pediatric nursing by presenting a prediction resource that can be used to minimize possible vascular traumas, and by favoring the option of adjuvant resources that facilitate the PIP in pediatrics. Assessment studies of the pediatric venous network are suggested, using the DIVA score as a tool, in other contexts, including external clinical validity, measuring equivalence and reproducibility analyses.
